# Improving Recognition and Reporting of Malignant Central Airways Obstruction on CT: The Need for Standardized Terminology and Structured Reporting

**DOI:** 10.7759/cureus.101897

**Published:** 2026-01-20

**Authors:** Catharine Pearce, Daniel Crowle, Richard Riordan, Adrian Marchbank, Cyrus Daneshvar

**Affiliations:** 1 Department of Respiratory Medicine, University Hospitals Plymouth NHS Trust, Plymouth, GBR; 2 Department of Radiology, University Hospitals Plymouth NHS Trust, Plymouth, GBR; 3 Department of Thoracic Surgery, University Hospitals Plymouth NHS Trust, Plymouth, GBR

**Keywords:** central airways obstruction, interventional pulmonology, lung cancer, oncology emergencies, radiology reporting, structured reporting, trigger words

## Abstract

Background

Malignant central airways obstruction (CAO) is a clinically significant complication of lung cancer that can lead to severe morbidity if not promptly recognized and is frequently under-reported on CT.

Methodology

All new annual lung cancer baseline CT scans were retrospectively reviewed as part of an audit process in 2014, 2019, and 2020. CT scans were assessed by the interventional pulmonology team. Patients with radiological CAO, defined as obstruction >50% in the trachea, main bronchi, or bronchus intermedius, were assessed. Narrative reports were separated into the main text section, conclusion, and addendum sections. Key phrases describing CAO were then extracted for further analysis. Analysis was performed in R Studio, tidytext, and wordcloud packages.

Results

In total, 140/1,096 (13%) new diagnoses of lung cancer had severe CAO on admission. No difference was seen across the three time periods (45/342 (13%) in 2014, 43/370 (12%) in 2019, and 52/351 (15%) in 2020; *P*-valve = 0.448). Of these, 135/140 (96%) had available CT scan radiology reports. Patient performance status was >2 in 50/135 (37%).  The presence of CAO was reported on the index CT scan in 97/135 (72%) of cases. The dominant obstructive component was extrinsic in 70/135 (52%), with a median (interquartile range (IQR)) area loss due to obstruction of 86% (64%-100%). The words (including truncations) central, obstruction, and occlusion were used in 29%, 36%, and 16% of the main text reports, respectively. Extracted key phrases that described CAO contained a median of 16 (9.5-25) words. The phrase *central airways obstruction,* or *CAO,* was not used in any CT reports.

Conclusions

Unreported CAO on CT scans, with variable and non-standardized terminology when present, is a concern. Adopting a standardized, structured reporting approach and the refinement of trigger words may improve CAO reporting and recognition.

## Introduction

Malignant central airways obstruction (CAO) is an often-late manifestation of primary lung cancer and is seen in around 1 in 10 patients at first presentation [1,2]. Computed tomography (CT) of the neck and thorax is the recommended imaging modality for suspected CAO [3]. However, CAO goes unreported by radiologists 30% of the time [1,4]. In symptomatic, malignant, severe CAO, referral for urgent therapies, including therapeutic bronchoscopy and radiotherapy, may be required. The CT scan is both a diagnostic and interventional planning tool, not only demonstrating CAO but also allowing for therapeutic procedural planning [5]. Patients with CAO have a poorer prognosis, and delays in recognition may impact the quality of life [1,3].

To improve reporting and downstream actions for patients with CAO, the identification of key phrases that initiate predefined responses may be of value. In aviation, standardized trigger words (e.g., *Mayday*, *Pan-pan*) immediately signal the need for emergency assistance, illustrating how concise, universally understood terminology can prompt timely and appropriate responses [6].

In medicine, trigger mechanisms extend beyond verbal cues to include structured thresholds or signals that prompt clinical response, such as early warning scores. Trigger-based approaches have also been used to detect adverse drug reactions, where targeted review of intensive care unit drug charts incorporating predefined trigger terms was shown to improve efficiency and case identification [7]. Similarly, in patients with subsequently confirmed cardiac arrest, emergency calls were reviewed to ascertain whether there were trigger words stated by callers, with the intention of improving the specificity of out-of-hospital cardiac arrest recognition [8]. No consistent trigger words were identified, highlighting the challenge untrained members of the public face in describing medical emergencies compared with the structured recognition and terminology used by trained professionals such as radiologists. This comparison underscores designing communication and recognition systems that support non-experts in high-stakes situations.

Stating the presence of CAO in radiological reports is a key prompt for referral to intervention, akin to that in spinal cord compression or superior vena cava obstruction (SVCO). We, therefore, explored the wording of reports in patients with known CAO.

The content of this article was originally presented as a poster at the 22nd British Thoracic Oncology Group (BTOG) annual conference, April 17-19, 2024 [9].

## Materials and methods

Cohort

This retrospective review of malignant CAO was conducted at University Hospital Plymouth, a large tertiary referral and teaching hospital in Devon, United Kingdom, serving a local secondary care population of approximately 475,000 and providing specialist services to a wider regional population of nearly two million across Devon, Cornwall, and surrounding areas [10].

We used the index staging thoracic CT scan reports of patients diagnosed with lung cancer to record the presence or absence of CAO over three time periods. These time periods included an index audit in 2014 [1], which examined the prevalence and local management of CAO in newly diagnosed lung cancer patients, with subsequent audits conducted in 2019 and 2020 to assess the impact of a dedicated CAO service [11].

Defining CAO

In brief, a focused review of the central airways was performed by two interventional pulmonologists to identify and determine the prevalence of severe CAO. The CT scans were double read, with each pulmonologist independently reviewing the images whilst blinded to the other reviewer’s assessment and to the original radiology reports. Severe CAO was defined as a reduction in the area of the central airway by >50% based on cross-sectional 2D estimates, as this degree of narrowing is generally associated with clinically significant airflow limitation and symptom development. The contralateral equivalent airway was used for comparison where necessary. In reports where CAO was considered unreported, scans were reviewed by a dedicated thoracic radiologist. 

Word analysis

Next, the wording of CT scan reports was manually extracted by human reviewers and entered in text format into an Excel spreadsheet. Radiology reports in the United Kingdom are typically structured into a main text section, which provides a detailed description of findings, and a conclusion section, summarizing key impressions. In some cases, an addendum is appended, often following a multi-disciplinary team (MDT) discussion or additional review by a senior and/or specialist radiologist. 

For our word analysis, the reports were therefore separated into these sections (main report text, conclusion, and any addenda) to allow focused extraction and evaluation of terminology. When present, the specific phrase within the report that the pulmonologist judged to describe the presence of CAO was manually identified and isolated for further analysis. Keywords/terms to search for were identified after discussion with interventional pulmonologists. Word analysis was performed in RStudio (version 2023.6.1.524), using tidytext and wordcloud work packages [12,13]. 

Statistical analysis

Proportions are described as several denominators (%), and comparisons are made using the chi-squared test. A *P*-value of <0.05 was considered significant. Multiple comparisons were accounted for using a Bonferroni correction.

Ethics approval

This study formed part of a local service evaluation to determine the local burden of disease and patient outcomes; formal ethics approval was not required. The purpose of the evaluation was to inform service organizations, management pathways, performance monitoring, and the development of audit standards. 

## Results

In total, 140/1,096 (12.8%; 95% confidence interval (CI) 10.8%-14.8%) new diagnoses of lung cancer had severe CAO on admission. No difference was seen across the three time periods (45/342 (13%) in 2014, 43/370 (12%) in 2019, and 52/351 (15%) in 2020; *P*-valve = 0.448). Of these, 135/140 (96%) had available CT scan radiology reports.

Patients were diagnosed during an emergency admission in 65/135 (48%) cases, and the World Health Organization Eastern Cooperative Oncology Group (WHO ECOG) performance status was 3-4 in 50/135 (37%). The dominant obstructive component, as determined by the interventional pulmonologist, was extrinsic (compression of the airway from outside the lumen) in 70/135 (52%), with a median (interquartile range) airway area loss due to obstruction of 86% (64%-100%). The presence of CAO was reported by radiologists on the index CT scan in 97/135 (72%) of cases. Notably, the observed prevalence of extrinsic obstruction is higher than previously reported. This may reflect the larger sample size in the present study, as earlier estimates (17/45, 38%) had a wide 95% CI (24%-53%); the current findings fall toward the upper limit of that interval, potentially indicating an evolving pattern of disease [1]. 

**Figure 1:**
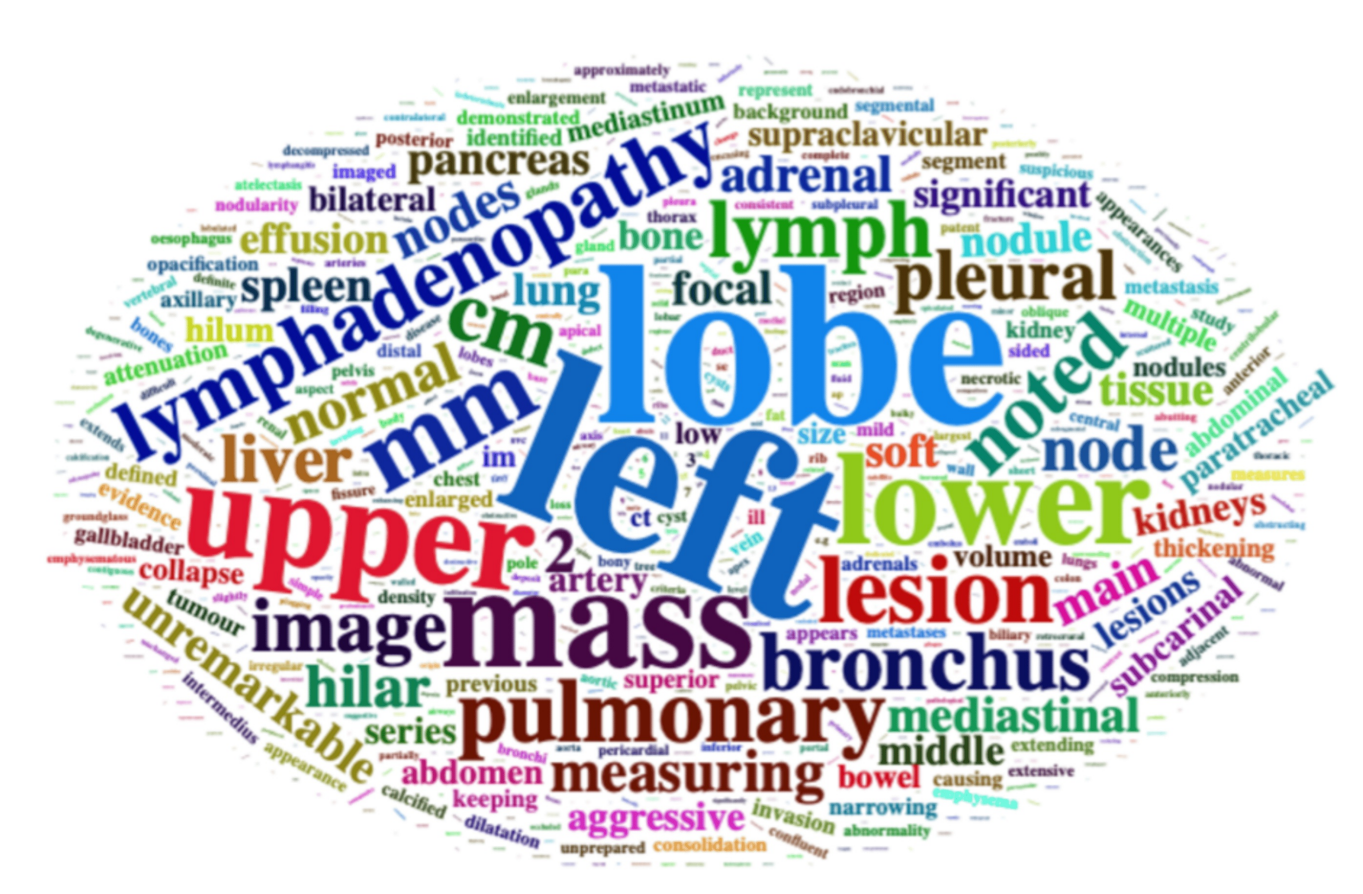
Word cloud of terms used in the main text of CT reports with CAO present. Image credit: Created by Cyrus Daneshvar using wordcloud [13]. CAO, central airways obstruction

Keywords (including truncations) *central*, *obstruction*, and *occlusion* appeared in 39/135 (29%), 49/135 (36%), and 15/135 (16%) of main text CT reports, respectively (Table 1, Figure 1). In the conclusion section, *obstruction *was the most commonly used term, but appeared in only 20/135 (15%) of reports, while narrow was used in 49/135 (36%) of main text reports and 11/135 (8%) of conclusions. Considering the full CT report (main text and conclusion), *obstruction *and *occlusion* were the most frequently used terms (55/135, 41% and 40/135, 30%, respectively), increasing further when addendum sections were included (67/135, 50% and 46/135, 34%, respectively).

**Table 1:** Keywords (including truncations) in CT reports where CAO present. ^a^Including truncations. *P < 0.005. **P < 0.05. CAO, central airways obstruction

Keyword^a^	All reports	Reported CAO	Unreported CAO
	*n* (%), *N* = 135	*n* (%), *N* = 96	*n* (%), *N* = 39
Main text/conclusion			
Blocked	0 (0%)	0 (0%)	0 (0%)
Bronchus*	122 (90%)	93 (97%)	29 (74%)
Central	41 (30%)	27 (28%)	14 (36%)
Compressed	63 (47%)	49 (51%)	14 (36%)
Emergency	0 (0%)	0 (0%)	0 (0%)
Endobronchial	19 (14%)	16 (17%)	3 (8%)
Narrow**	50 (37%)	42 (44%)	8 (21%)
Obstruction	55 (41%)	44 (46%)	11 (28%)
Occluded	40 (30%)	29 (30%)	11 (28%)
Stenosis	11 (8%)	7 (7%)	4 (10%)
Stent	31 (23%)	26 (27%)	5 (13%)
Urgent	16 (12%)	12 (13%)	4 (10%)
Focused phrase			
Blocked	0 (0%)	0 (0%)	0 (0%)
Bronchus*	108 (80%)	87 (91%)	21 (54%)
Central	2 (1%)	2 (2%)	0 (0%)
Compressed**	17 (13%)	16 (17%)	1 (3%)
Emergency	0 (0%)	0 (0%)	0 (0%)
Endobronchial	12 (9%)	12 (13%)	0 (0%)
Narrow**	35 (26%)	31 (32%)	4 (10%)
Obstruction	31 (23%)	26 (27%)	5 (13%)
Occluded	30 (22%)	23 (24%)	7 (18%)
Stenosis	8 (6%)	5 (5%)	3 (8%)
Stent	1 (1%)	1 (1%)	0 (0%)
Urgent	0 (0%)	0 (0%)	0 (0%)
Anywhere (including addendum)			
Blocked	0 (0%)	0 (0%)	0 (0%)
Bronchus	132 (98%)	96 (100%)	36 (92%)
Central	45 (30%)	30 (31%)	15 (38%)
Compressed**	65 (48%)	50 (52%)	15 (38%)
Emergency	0 (0%)	0 (0%)	0 (0%)
Endobronchial	25 (19%)	20 (21%)	5 (13%)
Narrow**	59 (44%)	48 (50%)	11 (28%)
Obstruction	67 (50%)	51 (53%)	16 (41%)
Occluded	46 (34%)	33 (34%)	13 (33%)
Stenosis	12 (9%)	7 (7%)	5 (13%)
Stent	46 (34%)	36 (38%)	10 (26%)
Urgent	17 (13%)	13 (14%)	4 (10%)

Notably, in cases classified as unreported CAO, the use of terms such as *obstruction*, *occluded*, or *narrowed* was most often used by radiologists to describe abnormalities outside the central airways, including lobar or segmental bronchial obstruction, vascular occlusion, or extrinsic compression of non-central structures. This interpretation is supported by the marked reduction in the use of these terms within the focused phrases extracted by pulmonologists to specifically describe central airways findings.

The use of the term *central* in reports classified as unrecognized CAO did not relate to CAO and referred to other anatomical locations rather than to clinically significant airway compromise.

The word *stent* was used 31 times in the main text body and conclusion, increasing to 46 with the addendum, suggesting a subsequent review at the lung cancer multidisciplinary meeting. The term *stent* was considered a recommendation for the airways in only one patient (reflected in the focused phrase). Other uses of the word *stent* in the additional reports were unrelated to CAO. The term *emergency* was not used in any cases. 

**Figure 2:**
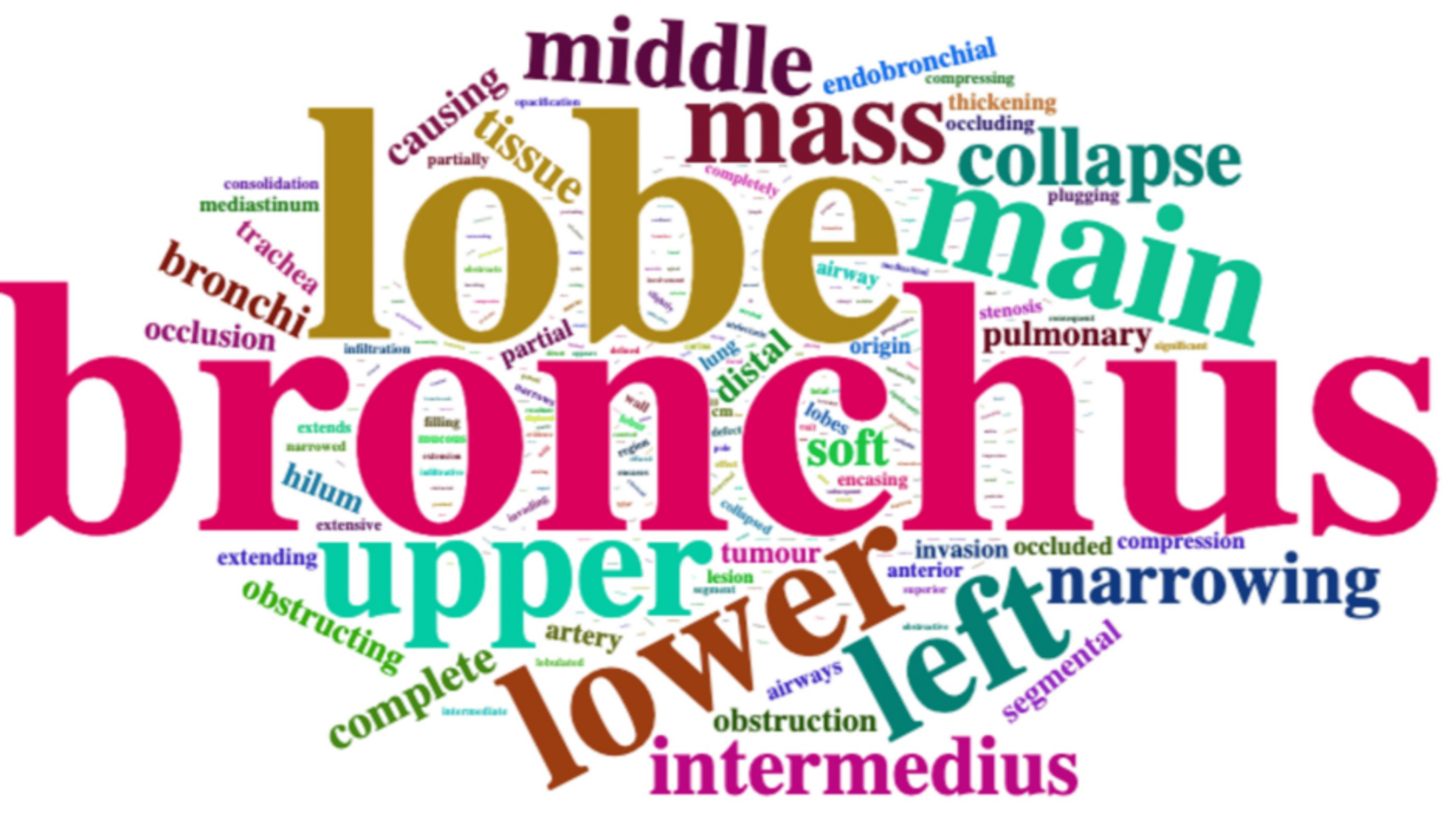
Word cloud of keywords describing CAO. Image credit: Created by Cyrus Daneshvar using wordcloud [13]. CAO, central airways obstruction

Within the extracted key phrases that described CAO, the phrases contained a median of 16 (9.5-25) words. The commonest word was bronchus (used 152 times) (Figure 2). The word *endobronchial *was used 12 times. Keywords most commonly used included *narrowing* (*n* = 36, 27%), *obstructing*/*obstruction* (*n* = 29, 21%), *occlusion*/*occluded* (*n* = 34, 25%), and *compressed* (*n* = 7, 5%). The word *complete *was used 21 times.

The phrase complete* occlu* was used six times, complete* obstruct* twice, and complete* collapse* on five occasions. The phrase *central airways obstruction* or *CAO* was not used in any CT reports.

## Discussion

CAO is an emergency presentation in lung cancer that requires prompt clinical escalation. In our study, we found that CAO was not reported in 28% of cases. Key phrases describing CAO were inconsistently used in radiology reports. Furthermore, additional keywords that clinicians would consider important for prompt recognition, such as obstruction and occlusion, were infrequently used. 

This gap in standardized reporting may contribute to delayed recognition and escalation to therapeutic management, potentially increasing patient morbidity. The reliance of treating clinicians on the radiology report as the definitive source of imaging interpretation highlights the requirement for structured reporting to ensure that significant findings, such as CAO, are consistently recognized and acted upon, especially in the context of known variability in free-text radiology reporting [14,15]. Furthermore, words and phrases used in radiology may be interpreted differently by radiologists and referring clinicians; ambiguous language in reports can affect clarity and clinical interpretation [16]. There is a particular risk in non-specialist centers where the reviewing clinician may not be an interventional pulmonologist, leading to under-recognition and a wide variation in care. This additionally emphasizes the importance of dedicated CT scan review in clinics, rather than relying on the reports alone.

By contrast, SVCO is a typically well-recognized phrase, with radiologists often communicating the severity, underlying cause, and potential clinical consequences of the obstruction in their reports [17]. The greater consistency in SVCO reporting likely reflects both heightened clinical awareness and the use of structured reporting templates. Clear and standardized reporting of SVCO has direct clinical implications, influencing time to treatment, symptom resolution, and the prevention of serious complications. This comparison highlights an opportunity to apply similar approaches to CAO, ensuring that potentially life-threatening airway obstruction is communicated consistently and unambiguously. 

The European Society of Radiology (ESR) endorses the use of structured reporting templates to improve clarity and reproducibility in radiology practice, thereby informing institutional policy, quality assurance processes, and the development of standardized reporting requirements [18]. Applying such frameworks to CAO, alongside refinement of trigger words, could facilitate earlier recognition and more effective clinical management; this could be evaluated through a pilot implementation or prospective audit. Structured reports may be especially valuable in district general hospitals or centers lacking onsite airway specialists, where clinicians depend primarily on radiology reports to recognize and manage CAO. Additionally, adopting standardized terminology could enable integration with automated tools, such as natural language processing algorithms, to flag potential CAO cases and further support timely clinical decision-making. 

Addressing this challenge requires coordinated work by key stakeholder groups. Based on our findings, we suggest adopting standardized descriptive terms, such as CAO, to describe clinically significant disease and improve clarity and consistency in radiology reporting. Embedding these terms in structured reports, combined with automated flagging of high-risk cases, could facilitate earlier recognition and more timely clinical intervention. Stakeholder collaboration would also help develop a shared understanding of treatment implications. Whilst these proposals serve as an initial framework, formal refinement and endorsement by multidisciplinary experts will be essential to ensure feasibility and widespread adoption, thereby helping to reduce the gap of unreported or under-reported scans [1-3].

Limitations

This study was conducted at a single tertiary referral center with a retrospective review of reports. The sample size of 135 patients provides insights, but may limit the generalizability of the findings to other centers or broader populations. The variability in radiologist expertise and individual reporting styles may have influenced the terminology used to describe CAO, introducing potential reporting bias. For example, 23% of focused phrases used the term *obstruction*, whereas *compressed* appeared in 13% of reports, highlighting the lack of consistency in descriptive language. While our findings likely reflect broader trends in non-specialist reporting, variability in factors such as referral completeness, image quality, and reporting environments globally suggests that the transferability of these results to other institutions or healthcare settings, with differing reporting cultures, warrants further investigation [19]. 

Although a thoracic radiologist verified unreported CAO cases, and agreement with the interventional radiologists was generally high, interobserver agreement was not formally quantified. A limitation of this study is that it focuses on the presence and frequency of key phrases in radiology reports, rather than the downstream clinical impact of reporting variation, such as time to diagnosis or intervention. Future work should prioritize assessing these outcomes, potentially through prospective audits or linkage with electronic health records, to evaluate how reporting practices influence patient care and the timeliness of management. Additionally, the extraction of narrative phrases relied on text-mining approaches, which may not capture all relevant descriptions, particularly if the language was ambiguous or non-standardized. To mitigate this, all reports were manually reviewed by the pulmonologists, allowing verification and contextual validation of the key phrases identified by the automated extraction. 

Despite these limitations, the findings highlight consistent gaps in CAO description and underscore the potential benefits of structured reporting and standardized terminology. Future multi-center studies could help validate these observations and assess the impact of structured reporting on patient outcomes. 

## Conclusions

Malignant CAO remains under-recognized in CT reports, with inconsistent terminology and absence of reporting in 28% of cases. Structured reporting templates for CAO could standardize communication and enhance patient safety. Structured reporting templates may also support quality improvement initiatives, including accelerated referral pathways, reduced time to bronchoscopy, and optimized timing of treatment. Prospective studies evaluating whether structured reporting and targeted trigger words lead to faster recognition, earlier intervention, and improved clinical outcomes, such as shorter time to multidisciplinary discussion and more timely treatment initiation, would be particularly valuable. CAO remains an understudied patient cohort, associated with poor prognosis and with few evidence-based treatments. Accurate reporting and prompt recognition are crucial for reducing diagnostic delays and ensuring equitable care for patients with CAO.

## References

[1] Daneshvar C, Falconer WE, Ahmed M (2019). Prevalence and outcome of central airway obstruction in patients with lung cancer. BMJ Open Respir Res.

[2] Ivanick NM, Kunadharaju R, Bhura S (2024). Epidemiology and survival of malignant central airway obstruction in lung cancer identified on cross-sectional imaging. J Bronchology Interv Pulmonol.

[3] Harris K, Alraiyes AH, Attwood K, Modi K, Dhillon SS (2016). Reporting of central airway obstruction on radiology reports and impact on bronchoscopic airway interventions and patient outcomes. Ther Adv Respir Dis.

[4] Powers RE, Schwalk AJ (2023). Overview of malignant central airway obstruction. Mediastinum.

[5] Oberg C, Folch E, Fernando Santacruz J (2018). Management of malignant airway obstruction. AME Med J.

[6] Federal Aviation Administration (2025). Federal Aviation Administration. Aeronautical Information Manual (AIM): Chapter 6 - Emergency Procedures [Internet]. Washington, D.C.: FAA; [cited 30 January 2025]. Available from: https://www.faa.gov/air_traffic/publications/atpubs/aim_html/chap6_section_3.html#:~:text=Transmit%20a%20distress%20or%20urgency,Aircraft%20identification%20and%20type. Aeronautical Information Manual: Chapter 6, Section 3 — Distress, Urgency, and Safety Signals.

[7] Kane-Gill SL, MacLasco AM, Saul MI (2016). Use of text searching for trigger words in medical records to identify adverse drug reactions within an intensive care unit discharge summary. Appl Clin Inform.

[8] Tamminen J, Lydén E, Kurki J, Huhtala H, Kämäräinen A, Hoppu S (2020). Spontaneous trigger words associated with confirmed out-of-hospital cardiac arrest: a descriptive pilot study of emergency calls. Scand J Trauma Resusc Emerg Med.

[9] Pearce C, Crowle D, Thorley R (2025). Reporting of central airways obstruction. Lung Cancer.

[10] (2025). University Hospitals Plymouth NHS Trust: Who we care for. https://www.plymouthhospitals.nhs.uk/about-us/.

[11] Zaki I, Arooj P, Crowle D (2022). P102 The impact of a dedicated interventional team in the management and outcome of central airways obstruction in lung cancer patients. Thorax.

[12] Silge J, Robinson S (2016). tidytext: Text mining and analysis using tidy data principles in R. J Open Source Software.

[13] Fellows I. wordcloud [Internet (2025). worldcloud. Github.

[14] Urbania TH, Dusendang JR, Herrinton LJ (2020). Standardized reporting and management of suspicious findings on chest CT imaging is associated with improved lung cancer diagnosis in an observational study. Chest.

[15] Huesch MD, Cherian R, Labib S, Mahraj R (2018). Evaluating report text variation and informativeness: natural language processing of CT chest imaging for pulmonary embolism. J Am Coll Radiol.

[16] Lee B, Whitehead MT (2017). Radiology reports: What you think you’re saying and what they think you’re saying. Curr Probl Diagn Radiol.

[17] Silverstone L (2025). Radiopedia: Superior vena cava obstruction. https://radiopaedia.org/articles/superior-vena-cava-obstruction?lang=gb.

[18] (2018). ESR paper on structured reporting in radiology. Insights Imag.

[19] Gormly KL (2025). Improving radiology reporting locally and globally: who, how, and why?. Br J Radiol.

